# Supplementary material for: Chemical Protein Crosslinking-Coupled Mass Spectrometry Reveals Interaction of LHCI with LHCII and LHCSR3 in Chlamydomonas reinhardtii
Source: Plants (Basel). 2024 Jun 13;13(12):1632. doi: 10.3390/plants13121632 (PMC11207971; doi:10.3390/plants13121632)
Supplement: Supplementary file 1 [file plants-13-01632-s001.zip › Supplemental Figures.pdf]

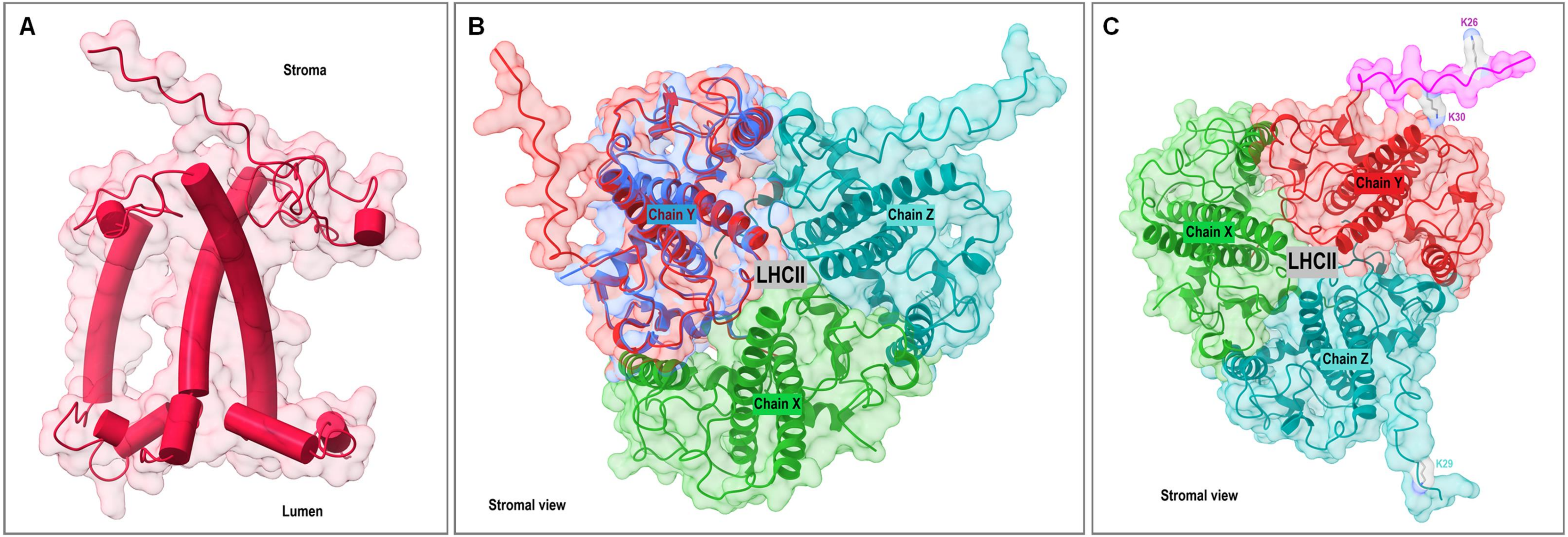

Supplemental Figure 1: (**A**) Model structure of LHCBM3 (Chain Y) as predicted by AlphaFold2 (pLDDT 91.9), with the missing loop containing the crosslinked lysine residues being resolved. The helices are shown as tubes. (**B**) Superposition of the predicted LHCBM3 model with LHCII trimer 1 (PDB ID: 7E0J). The newly modelled LHCBM3 is shown in red while the LHCBM3 from 7E0J is shown in marine. (**C**) LHCII trimer 1 with the newly modelled LHCBM3. The crosslinked residues (K26 and K30) are shown as sticks.

A

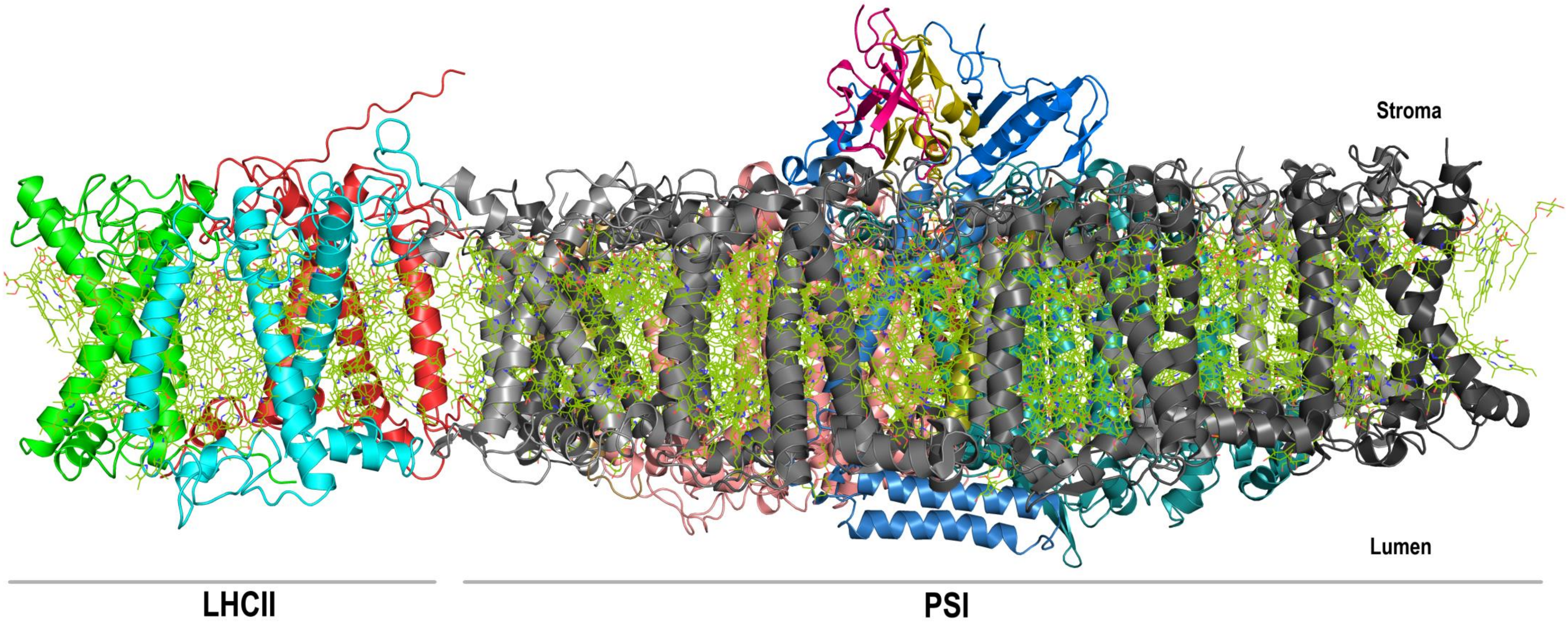

Supplemental Figure 2: Membrane view of the docking of LHCII (PDB ID: 7E0J with newly modelled Chain Y) to PSI-LHCI (PDB ID: 7ZQC) at two potential positions (**A** and **B**) based on crosslinking data and the density observed in negatively stained particles (Steinbeck et al., 2018) . PSI core and LHCII subunits are in color, LHCI are in grey.

A

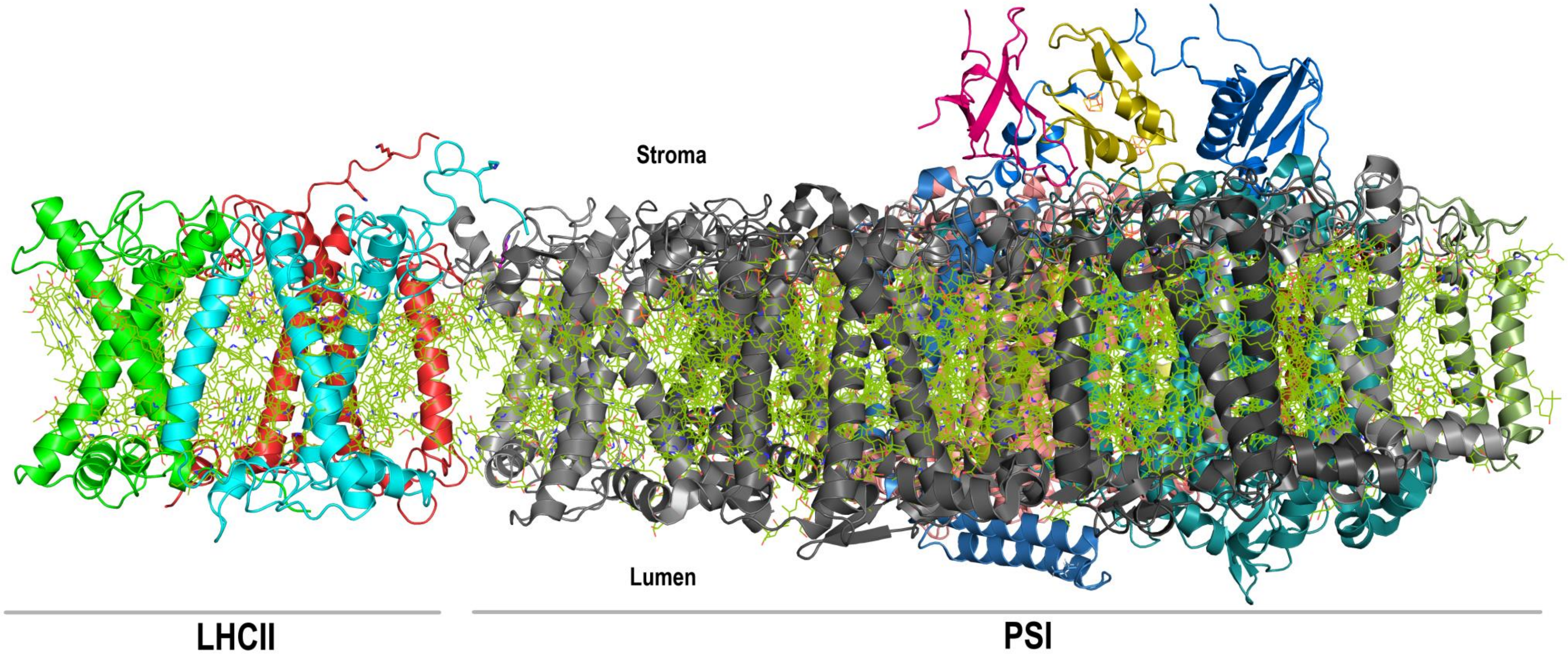

Supplemental Figure 2: Membrane view of the docking of LHCII (PDB ID: 7E0J with newly modelled Chain Y) to PSI-LHCI (PDB ID: 7ZQC) at two potential positions (**A** and **B**) based on crosslinking data and the density observed in negatively stained particles (Steinbeck et al., 2018) . PSI core and LHCII subunits are in color, LHCI are in grey.

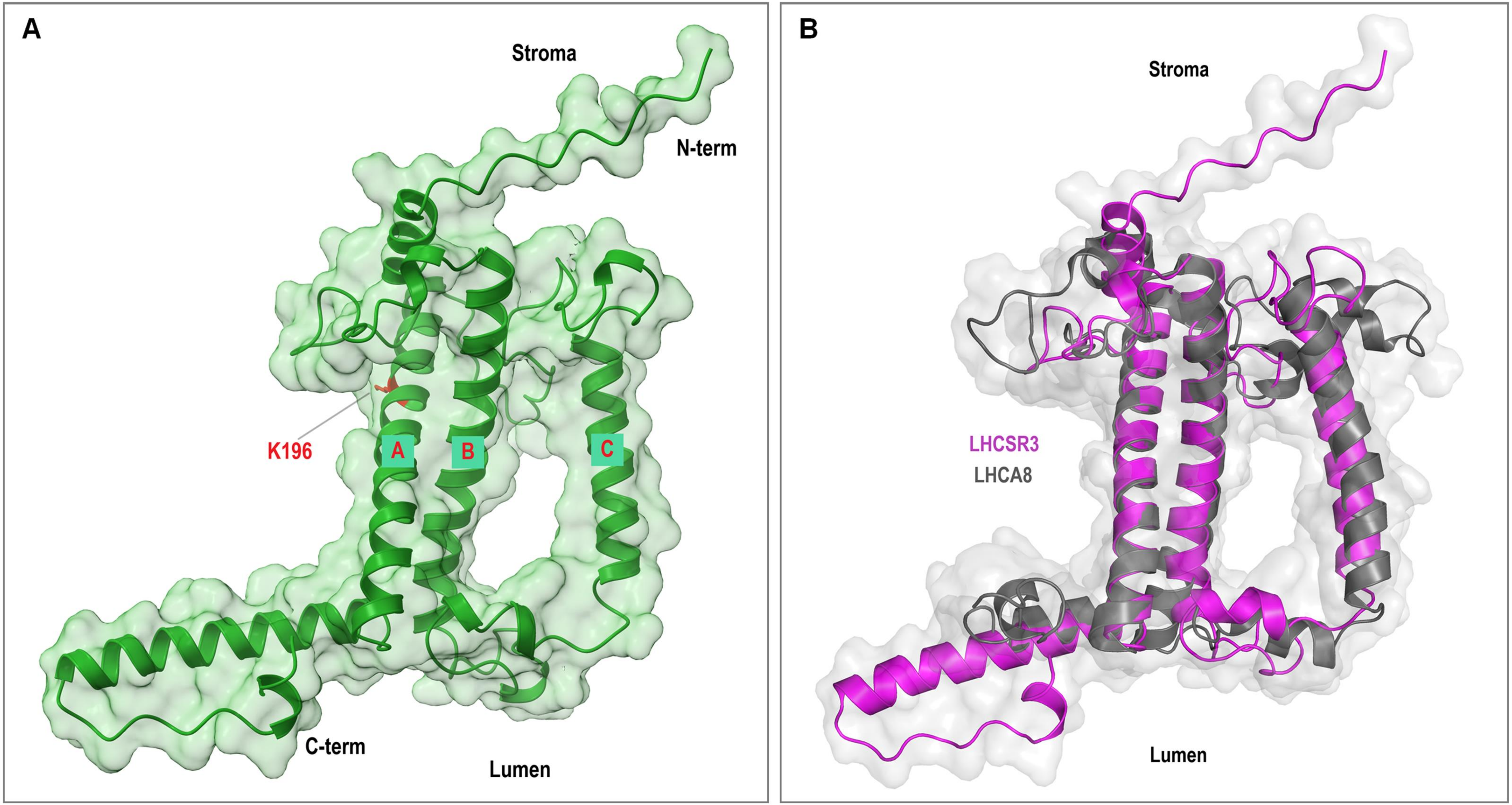

Supplemental Figure 3: (**A**) Model structure of LHCSR3 as predicted by AlphaFold2 (pLDDT 85.6). The crosslinked residue (K196) is represented as stick. (**B**) Superposition of LHCSR3 with LHCA8 from *C. reinhardtii* (PDB ID: 7ZQC).

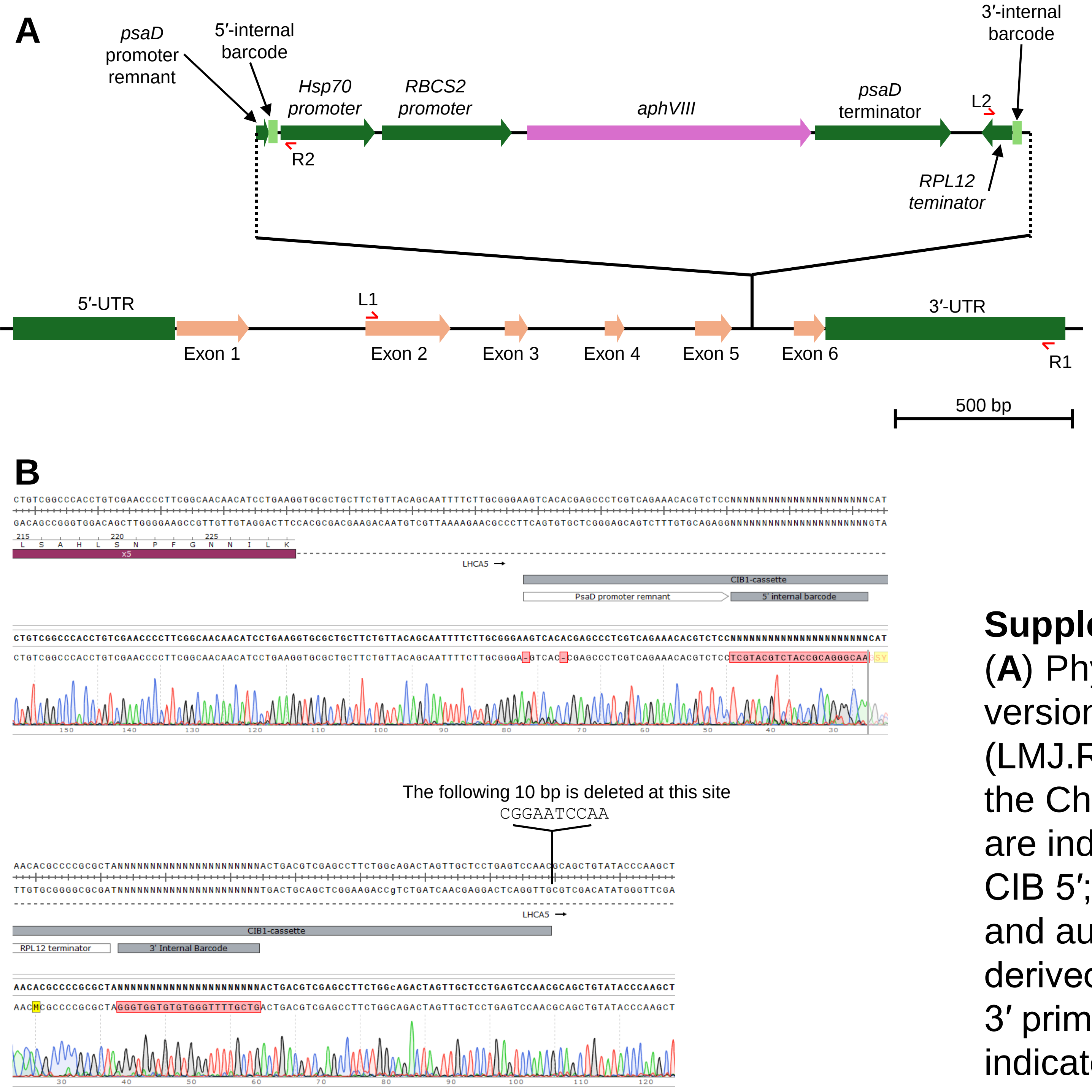

Supplemental Figure 4: CIB cassette insertion in *lhca5* (**A**) Physical map of *LHCA5* (Cre10.g425900.t1.1, in version 5.6) in the *lhca5* insertional mutant strain (LMJ.RY0402.044057). Gene annotations were based on the Chlamydomonas genome version 5.6. Primer positions are indicated as L1: Lhca5 SGP1; R1: Lhca5 SGP2 ; R2: CIB 5′; L2: CIB 3′. (**B**) Chromatograms of DNA sequencing and automatically annotated. Upper and lower panels are derived from the sequencing reaction using CIB 5′ and CIB 3′ primers, respectively. The 10 bp deletion site is indicated.

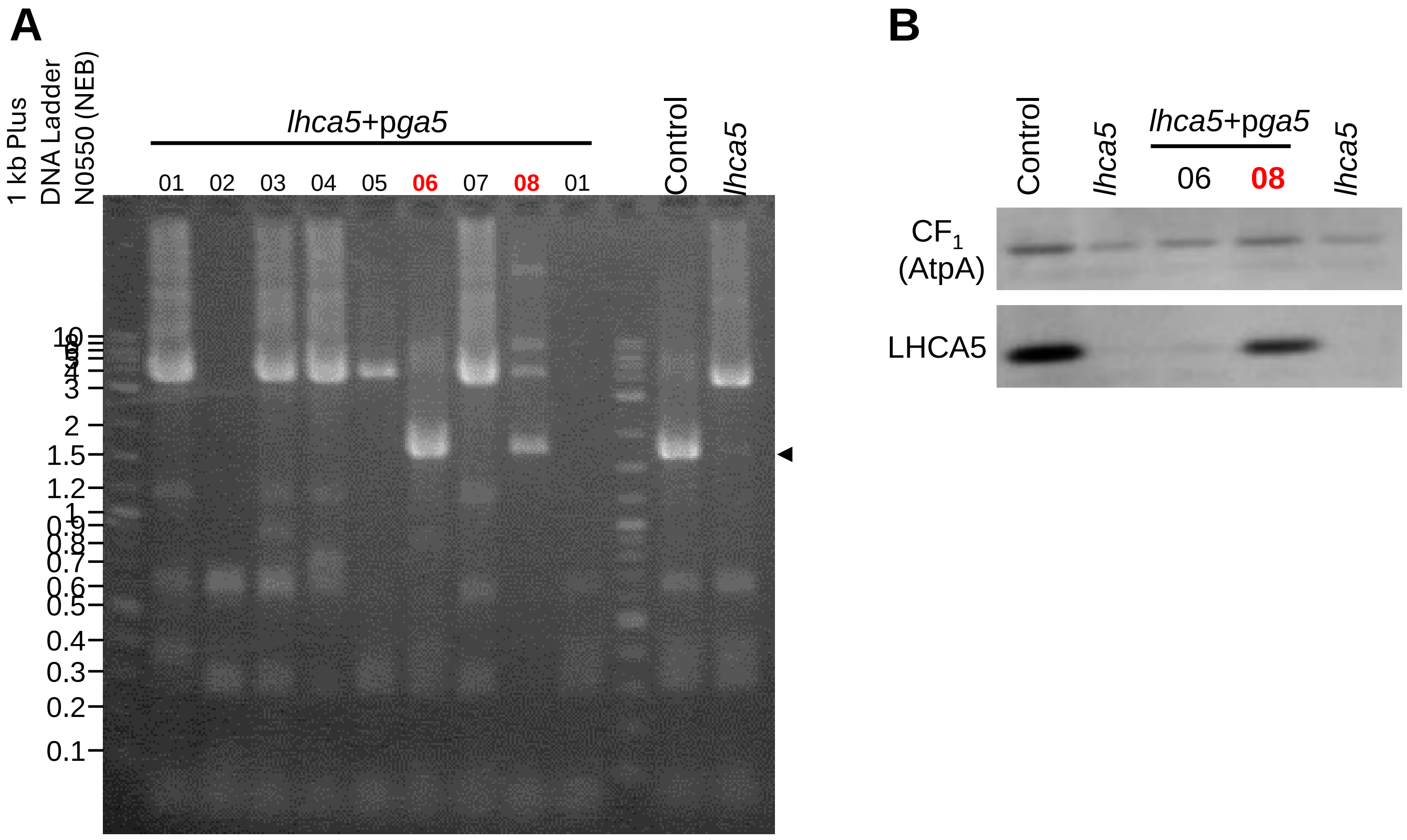

Supplemental Figure 5: Complementation of LHCA5 in *lhca5* (**A**) Screening of transformants for *LHCA5*. PCR products generated with Lhca5_SGP1/2 primers were separated by agarose electrophoresis with TAE buffer and visualized with a UV transilluminator after staining with EtBr. The migration position of the PCR product from wild type *LHCA5* is indicated (arrowhead). PCR templates as labelled on top: Individual clone numbers of transformants (*lhca5*+p*ga5*), control (CLiP library control strain) and the recipient strain (*lhca5*). Clones #6 and #8 were identified as candidates to verify LHCA5 protein accumulation by immunoblotting. (**B**) Immunoblotting of whole cell samples separated by SDS-PAGE, probed with CF1 (anti-AtpA) or LHCA5 antibodies.

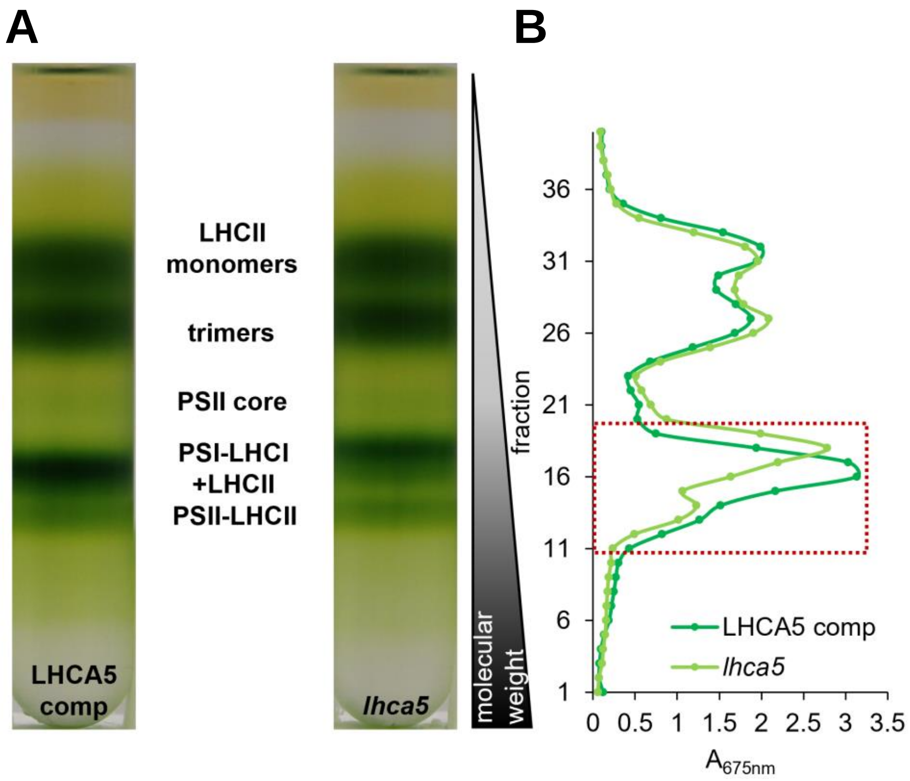

Supplemental Figure 6: (**A** and **D**) Sucrose density gradients of α-DDM solubilized thylakoids (~250 µg chl) isolated from the *lhca5* complemented strain and the *lhca5* insertional mutant. Cells were shifted to low CO_2 (medium without carbon source) for 40 h and high light (150 µmol m^{-2} s^{-1}) for 24 h prior to the isolation. (**B** and **E**) Absorption profile of SDG fractions at 675 nm. SDG fractions selected for SDS-PAGE followed by immunoblotting are indicated by the red box. (**C** and **F**) Immunoblots against PSAA, PSBA, LHCSR3, LHCA5 and PSAD. Samples were loaded on equal volume. Peak fractions of PSII-LHCII, PSI-LHCI-LHCII and PSI-LHCI are indicated by red boxes.

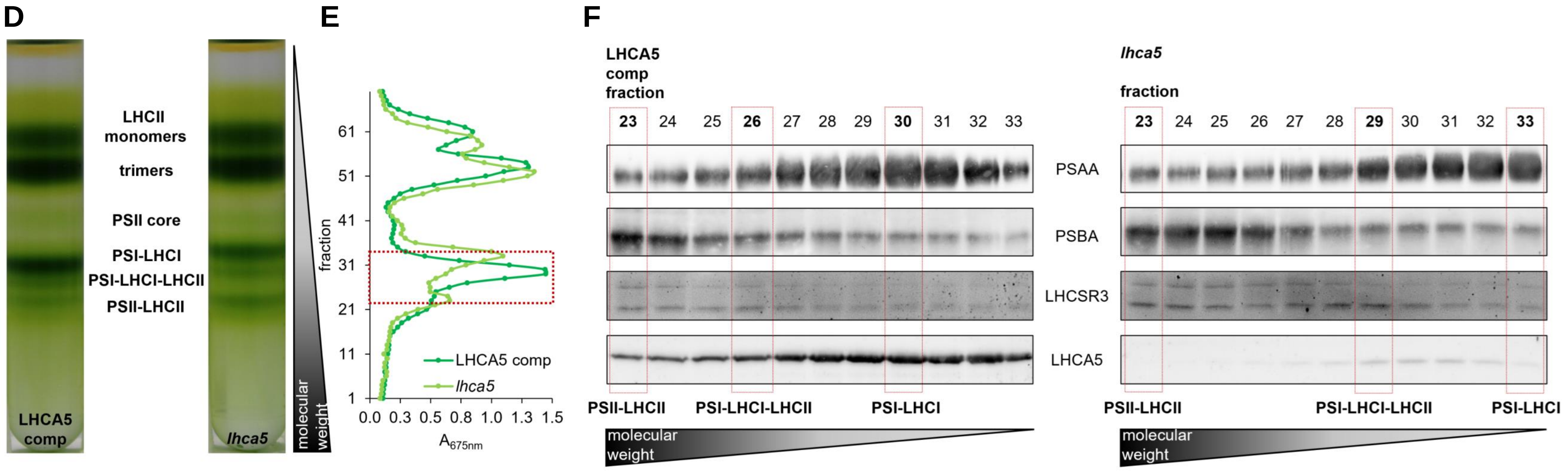

Supplemental Figure 6: (**A** and **D**) Sucrose density gradients of α-DDM solubilized thylakoids (~250 µg chl) isolated from the *lhca5* complemented strain and the *lhca5* insertional mutant. Cells were shifted to low CO_2 (medium without carbon source) for 40 h and high light (150 µmol m^{-2} s^{-1}) for 24 h prior to the isolation. (**B** and **E**) Absorption profile of SDG fractions at 675 nm. SDG fractions selected for SDS-PAGE followed by immunoblotting are indicated by the red box. (**C** and **F**) Immunoblots against PSAA, PSBA, LHCSR3, LHCA5 and PSAD. Samples were loaded on equal volume. Peak fractions of PSII-LHCII, PSI-LHCI-LHCII and PSI-LHCI are indicated by red boxes.
