# Supplementary material for: Chemical Protein Crosslinking-Coupled Mass Spectrometry Reveals Interaction of LHCI with LHCII and LHCSR3 in Chlamydomonas reinhardtii
Source: Plants (Basel). 2024 Jun 13;13(12):1632. doi: 10.3390/plants13121632 (PMC11207971; doi:10.3390/plants13121632)
Supplement: Supplementary file 1 [file plants-13-01632-s001.zip › Supplemental_Document_1.pdf]

Merox

```
ELEMENTS                                        //List of all elements with exact masses, maximum two
letters allowed first one must be upper case, second one must be lower case
 C;12.0
 D;2.01410178
 Ch;13.00335484
 F;18.9984032
 H;1.00782503
 Cl;34.9688527
 I;126.904468
 K;38.9637069
 N;14.003074
 O;15.99491462
 P;30.973762
 Br;78.9183376
 S;31.97207117
 Na;22.9897697
 T;3.01604927
 Si;27.9769265
 Proton;1.00727638
 Oh;17.99915961
 Nh;15.0001089
 Li;60.151223
 Fe;55.9349393
END
MINMASS=1000.0                                  //minimum mass for peptides / cross-links
MAXMASS=8000.0                                  //maximum mass for peptides / cross-links
PRECISIONMS=10.0                                //precision of MS1 matching in ppm
PRECISIONMS2=20.0                       //precision of MS2 matching in ppm or Da (see MS2UNIT)
MSRECALIBRATION=0.0                     //recalibrate the masses of MS1 signals by x ppm
MS2RECALIBRATION=0.0            //recalibrate the masses of MS2 signals by x ppm/Da (see MS2UNIT)
MS2UNIT=ppm                                     //Unit of MS2 comparison
SNRATIO=2.0                                     //signal-to-noise ratio
MAXRANGE=0.1                            //
MINCHARGE=2                                     //minimum charge of precursor ions to be considered
IGNORECHARGESTATE=0                     //Ignore charge state determined by conversion software (not
recommended!)
TESTCHARGES=3;4;5                       //Charges to test, if actualcharge state is ignored (separated by ;)
PROTONMASS=1.00727638                   //mass of protons
IONTYPE=by                                      //iontypes to be considered
DIGITSMSMS=3                            //number of digits presented in GUI for MS2 signals
DIGITSMS=3                                      //number of digits presented in GUI for MS1 signals
DIGITSDEVIATION=2                       //number of digits presented in GUI for mass deviation
MINPEPLENGTH=6                                  //minimum length of peptide sequences to consider
MAXPEPLENGTH=30                                 //maximum length of peptides to consider
MAXMISSEDCLEAVAGES=3                    //maximum number of misscleavages in total (for all proteases
sites combined.)
UNSPECIFICDIGEST=0                      //unspecific digest (0-Off, 1-On)
SEMIUNSPECIFICDIGEST=0                  //semiunspecific digest (0-Off, 1-On) only one side of the peptides
needs to arise from one of the defined preotease sites
```

UNIQUEPEPTIDESONLY=0 //only peptides that are unique within the provided database will be considered
L_EQUALS_I=0 //When determining unique peptides, Leucine is not differentiated from Isoleucine (0-Off, 1=On)
LOSSSETTING=2 //Consider losses of MS2 signals: 1 - No losses considered, 2 - neutral losses only of identified signals, 3 - neutral losses of all possible fragments
MAXLOSSES=1 //maximum number of neutral losses per fragment ion
INCLUDESPECIFIC=0 //include a specified loss for all fragment ions (see SPECIFICLOSS)
SPECIFICLOSS=CO2 //elemental composition of the specific loss (see INCLUDESPECIFICLOSS)
INCLUDESPECIFICPREC=0 //Include specific precursor loss defined for the cross-linker (e.g. N2)
SCOREDEPTH=4 //Depth of scoring e.g. number of mass-shifted spectra to compare to 0 - no scoring 1-4 increasing depth
SCORECUTOFF=50.0 //minimum score required to save candidate to result file
FDRCUTOFF=0.05 //maximum false discovery rate for cross-link identification
APPLYFDR=1 //calculate FDR and use as filter (0-Off, 1-On) recommended to be always on
ONLYSHOWBESTRESULTS=1 //Only save the best scoring candidate to the result file (0-Off, 1-On) if off, the candidates will be ranked for each spectrum by the score.
SAVEDECOYS=1 //Save decoy hits in the reuslt file and show them in the canduadate table
CONSECUTIVEPEPTIDES=1 //exclude consecutive sequences for type 2 cross-links (0-include, 1-exclude)
PRESCORE=1 //perform a prescoring to sort out bad matching spectra more quickly (0-Off, 1-On)
PRESCRLIMIT=0.1 //minimum coverage of total intensity of signals (excluding precursor region)
MINPEPSCORE=10 //mimimum score for individual peptides (only applies to RISEUP and proteome-wide mode)
MINPEPSCOREQUADRATIC=10 //mimimum score for individual peptides (used in quadratic mode)
MINFRAGMENTSPERPEPTIDE=3 // minimum number of fragments per peptide in a cross-link
DEISOTOPE=1 //perform deisotoping on MS2 spectra (0-Off, 1-On) recommended to be on. Turn off, if spectra are already deisotoped (charge information on signals will be lost then)
ANALYSISMODE=0 //0-Quadratic search space 1-RISE-Mode 2-ProteomeMode 3-RISEUP-Mode
MAXMISSINGFRAGMENTS=1 //Number of missing cross-linker fragments from a complete pattern to still be recognized by RISE- and RISEUP-mode
INCLUDEINTERNALFRAGMENTS=1 //include fragments in scoring matching linear ions from cross-linker fragmentation (Mass-modification = 0Da)
MIXEDTARGETDECOY=1 //Include mixed decoys, where only of the two peptides is from the decoy database. (0-Off, 1-On) Recommended to be always on.
DECOYTYPE=0 //Type of decoy database generation: (0-shuffle protein sequences but Keep protease sites, 1-shuffle protein sequences, 2-reverse protein sequences)
DECOYLEVEL=2 //Expert option! recommend to use 2! Basis of decoy database generation: (0-Only Fasta-entries labelled with decoy, 1-protein-level, 2-peptide-level)
DECOYIDENTIFIER=DEC_ //Prefix to fasta header defining decoy entries in the fasta file e.g.: >DEC_sp|P02769|ALBU_BOVIN Serum albumin OS=Bos taurus OX=9913 GN=ALB PE=1 SV=4
PERFORMDECOY=1 //Run decoy analysis in parallel (0-Off, 1-On) Recommended to be always on.
INCLUDECRAP=0 //Include a database of common contaminating proteins (cRAP) (0-Off, 1-On) Not recommended in Quadratic search-mode (StavroX-mode)

INTRAPEPTIDAL=0 //Search for intrapeptidal (type 1) cross-links (0-Off, 1-On)
DEADEND=0 //Search for dead-end (type 0) cross-links (0-Off, 1-On)
CORRECTPRECURSORMASSES=0 //Correct wrongly assigned precursor masses (errors during format conversion) (0-Off, 1-On). For mzML and mzXML MS1 data is used to correct. For other file types, different precursor masses are checked with up to <N-VALUE> isotope shifts
N_VALUE=3 //maximum number of isotopshifts for precursor mass correction
D_VALUE=1.0033548 //mass difference of 13C and 12C used for precursor mass correction
SAVESPECTRATOTEMP=0 //spectra are saved to a temporary folder. (0-Off, 1-On) mzML and mzXML are always saved to temporary folder. For other file types this might reduce RAM-load, but takes more time.
IONTYPES //2 additional ion types can be defined here.
z';-NH;C
z'';-N;C
END
AMINOACIDS //definition of all amino acids with name;single-letter code;composition. Peptide and Protein termini are also defined
Alanine;A;C3H5NO
Cystein acetamide;B;C5H8N2O2S
Cysteine;C;C3H5NOS
Asparaginic acid;D;C4H5NO3
Glutaminic acid;E;C5H7NO3
Phenylalanine;F;C9H9NO
Glycine;G;C2H3NO
Histidine;H;C6H7N3O
Isoleucine;I;C6H11NO
Methionine oxidized;J;C5H9NO2S
Lysine;K;C6H12N2O
Leucine;L;C6H11NO
Methionine;M;C5H9NOS
Asparagine;N;C4H6N2O2
Proline;P;C5H7NO
Glutamin;Q;C5H8N2O2
Arginine;R;C6H12N4O
Serine;S;C3H5NO2
Threonine;T;C4H7NO2
Valine;V;C5H9NO
Tryptophane;W;C11H10N2O
Tyrosine;Y;C9H9NO2
HydrogenN-Terminal;[;H
HydroxyC-Teminlal;];OH
Alanine;a;C3H5NhO
Cystein acetamide;b;C5H8NNh1O2S
Cysteine;c;C3H5NhOS
Asparaginic acid;d;C4H5NhO3
Glutaminic acid;e;C5H7NhO3
Phenylalanine;f;C9H9NhO
Glycine;g;C2H3NhO
Histidine;h;C6H7Nh3O
Isoleucine;i;C6H11NhO
Methionine oxidized;j;C5H9NhO2S
Lysine;k;C6H12Nh2O

```
  Leucine;l;C6H11NhO
  Methionine;m;C5H9NhOS
  Asparagine;n;C4H6Nh2O2
  Proline;p;C5H7NhO
  Glutamin;q;C5H8Nh2O2
  Arginine;r;C6H12Nh4O
  Serine;s;C3H5NhO2
  Threonine;t;C4H7NhO2
  Valine;v;C5H9NhO
  Tryptophane;w;C11H10Nh2O
  Tyrosine;y;C9H9NhO2
  HydrogenN-Terminus;{;H
  HydroxyC-Teminlus;};OH
END
USEDCROSSLINKER=BS3/DSS-D0/D12                    //Cross-linker that is selected from the
following list.

CROSSLINKER=Formaldehyde(24)
  COMPOSITION=C2
  COMPHEAVY=
  SITE1=K{
  SITE2=KSTY{
  MAXIMUMDISTANCE=14.5
END

CROSSLINKER=BS3/DSS-D0/D12
  COMPOSITION=C8H10O2
  COMPHEAVY=C8D12O2-H2
  SITE1=K{
  SITE2=KSTY{
  MAXIMUMDISTANCE=23.8
  RETENTIONDIFF=40
  MODSITE1
    Pep;;0
  MODSITE2
    Pep;;0
END

CROSSLINKER=DSBU
  COMPOSITION=C9O3N2H12
  COMPHEAVY=
  SITE1=K{
  SITE2=KSTY{
  MAXIMUMDISTANCE=26.9
  CNL=C4H7NO
  DEADENDMOLECULE=H2O
  REPORTERIONS
    RBu;C9N2OH17
    RBuUr;C10N2O2H15
  MODSITE1
    Bu;C4NOH7;1
    BuUr;C5O2NH5;1
    Pep;;0
```

```
  MODSITE2
    Bu;C4NOH7;1
    BuUr;C5O2NH5;1
    Pep;;0
END

CROSSLINKER=DSAU
  COMPOSITION=C5N2O3H4
  COMPHEAVY=
  SITE1=K{
  SITE2=KSTY{
  MAXIMUMDISTANCE=23.0
  DEADENDMOLECULE=H2O
  MODSITE1
    Ac;C2NOH3;1
    AcUr;C3O2NH;1
    Pep;;0
  MODSITE2
    Ac;C2NOH3;1
    AcUr;C3O2NH;1
    Pep;;0
END

CROSSLINKER=Formaldehyde(12)
  COMPOSITION=C
  COMPHEAVY=
  SITE1=K{
  SITE2=KSTY{
  MAXIMUMDISTANCE=14.5
END

CROSSLINKER=DST
  COMPOSITION=C4O4H2
  COMPHEAVY=
  SITE1=K{
  SITE2=KSTY{
  MAXIMUMDISTANCE=18.65
  DEADENDMOLECULE=H2O
  MODSITE1
    Pep;;0
  MODSITE2
    Pep;;0
END

CROSSLINKER=EDC
  COMPOSITION=-H2O
  COMPHEAVY=
  SITE1=K{
  SITE2=DE}
  MAXIMUMDISTANCE=11.2
  DEADENDMOLECULE=H2O
  MODSITE1
    Pep;;0
```

```
  MODSITE2
    Pep;;0
END

CROSSLINKER=CDI
  COMPOSITION=CO-H2
  COMPHEAVY=
  SITE1=K{
  SITE2=KSTY{
  MAXIMUMDISTANCE=16.0
  DEADENDMOLECULE=H2O
  MODSITE1
    CO;CO-H2;1
    Pep;;1
  MODSITE2
    CO;CO-H2;1
    Pep;;1
END

CROSSLINKER=DSSO
  COMPOSITION=C6O3SH6
  COMPHEAVY=
  SITE1=K{
  SITE2=KSTY{
  MAXIMUMDISTANCE=23.5
  DEADENDMOLECULE=H2O
  MODSITE1
    A;C3OH2;1
    T;C3OSH2;1
    S;C3O2SH4;0
    Pep;;0
  MODSITE2
    A;C3OH2;1
    T;C3OSH2;1
    S;C3O2SH4;0
    Pep;;0
END

CROSSLINKER=SDA
  COMPOSITION=C5H6O
  COMPHEAVY=
  SITE1=K{
  SITE2=DE}
  MAXIMUMDISTANCE=16.0
  DEADENDMOLECULE=H2O
  MODSITE1
    Pep�+82u;C5H6O;1
    Pep�+100u;C5H8O2;1
    Pep�;;1
  MODSITE2
    Pep#;;1
END
```

```
CROSSLINKER=DC4
  COMPOSITION=C14O2N2H20
  COMPHEAVY=
  SITE1=K{
  SITE2=KSTY{
  MAXIMUMDISTANCE=32.5
  DEADENDMOLECULE=H2O
  REPORTERIONS
    R-DABCO;C10N2O2H19
  MODSITE1
    Fur;C4H4O;1
    DABCO;C10ON2H16;1
    Pep;;0
  MODSITE2
    Fur;C4H4O;1
    DABCO;C10ON2H16;1
    Pep;;0
END

CROSSLINKER=BS3/DSS
  COMPOSITION=C8H10O2
  COMPHEAVY=
  SITE1=KSTYksty{
  SITE2=KSTYksty{
  MAXIMUMDISTANCE=23.8
  MODSITE1
    Pep;;0
  MODSITE2
    Pep;;0
END

CROSSLINKER=BS2G
  COMPOSITION=C5H4O2
  COMPHEAVY=C5D4O2
  SITE1=K{
  SITE2=KSTY{
  MAXIMUMDISTANCE=19.8
  RETENTIONDIFF=40
  DEADENDMOLECULE=H2O
  MODSITE1
    Pep;;0
  MODSITE2
    Pep;;0
END
PROTEASE                                                  //List of protease sites
  {M?;1;;0
  R?;3;P;0
  K?;3;P;1
  {m?;1;;0
  r?;3;P;0
  k?;3;P;1
END
```

```
POSTRANSLATIONALMODIFICATION            //List of possible posttranslational modifications (peptide level, no
localization attempted)
END
VARMODIFICATION                                              //List of variable modifications (residue-level,
different localizations tested)
  m;j;3
  M;J;3
END
STATMODIFICATION                                    //List of static modifications (all residues will be modified)
END
```

XiSearch

```
####################
##Tolerances
## for matching the precursor information
tolerance:precursor:10ppm
## for matching fragments to the ms2-peaks
tolerance:fragment:20ppm


#####################
## how many cpus to use
## values smaller 0 mean that all avaiblable but the mentioned number will be used
## e.g. if the computer has 4 cores and UseCPUs is set to -1 then 3 threads are used for search.
## this is a bit relativated by the buffering, as buffers also use threads to decouple the input and output of the buffer.
## each thread will also have a small buffer between itself and the input and the output queue - but the overal cpu-usage of these should be smallish
UseCPUs:15


##============================
## Homobifunctional
## format :
## crosslinker:SymetricSingleAminoAcidRestrictedCrossLinker:Name:[name];MASS:[cross-linker mass];LINKEDAMINOACIDS:[list of possible cross-link targts];MODIFICATIONS:[list of associated modifications];decoy
## with:
##  Name:           A name of the cross-linker
##  MASS:           The mass of the cross-linker as  the difference between the mass of the two peptides and the mass of the mass of the two peptides when reacted with the cross-linker
##  LINKEDAMINOACIDS: A comma separated list of amino-acids that the cross-linker can react with. Additionaly nterm and cterm are accepted
##                Also amino-acids can get a ranking by defining a penelty (between 0 and 1) for them.
##                E.g. K(0),S(0.2),T(0.2),Y(0.2),nterm(0) means that K and the protein n-terminal are more likely to be cross-linked then S, T, or Y
## MODIFICATIONS:     a comma-separeted list defining related modifications
##                E.g. NH3,17.026549105,OH2,18.0105647
##                defines two modifications:
##                  NH3: that adds 17.026549105 to the mass of the cross-linker
##                  OH2: that adds 18.0105647 to the mass of the cross-linker
## LINAERMODIFICATIONS: same as MODIFICATIONS but will only be applied to linear peptides
## LOSSES:          a comma-separeted list defining crosslinker related losses
##                E.g. X,10,Y120
##                defines two losses:
##                  X: a loss of 10Da from the cross-linker
##                  Y: a loss of 120Da from the cross-linker
## STUBS:          a comma-separeted list defining crosslinker stubs for MS-cleavable cross-linker
##                E.g. A,54.0105647,S,103.9932001,T,85.9826354
##                defines three cross-linker stubs:
##                  A: with mass 54.0105647
##                  S: with mass 103.9932001
##                  T: with mass 85.9826354
```

##
additionally one can also define heterobifunctional cross-linker
crosslinker:AsymetricSingleAminoAcidRestrictedCrossLinker:Name:[name];MASS:[cross-linker mass];FIRSTLINKEDAMINOACIDS:[list of possible cross-link targts];SECONDLINKEDAMINOACIDS:[list of possible cross-link targts]
syntax is similare to homobifinctional crosslinker with two changes:
two sets of specificities FIRSTLINKEDAMINOACIDS and SECONDLINKEDAMINOACIDS and modifications cant be defined together with the cross-linker
##
##DSS
crosslinker:SymetricSingleAminoAcidRestrictedCrossLinker:Name:DSS;MASS:138.06807;LINKEDAMINOACIDS:K(0),S(0.2),T(0.2),Y(0.2),nterm(0)
##DSSheavy
#crosslinker:SymetricSingleAminoAcidRestrictedCrossLinker:Name:DSS12;MASS:150.1434;LINKEDAMINOACIDS:K(0),S(0.2),T(0.2),Y(0.2),nterm(0)
##BS3
##crosslinker:SymetricSingleAminoAcidRestrictedCrossLinker:Name:BS3;MASS:138.06807;LINKEDAMINOACIDS:K(0),S(0.2),T(0.2),Y(0.2),nterm(0)
#crosslinker:SymetricSingleAminoAcidRestrictedCrossLinker:Name:BS3;MASS:138.06807;LINKEDAMINOACIDS:K,nterm;MODIFICATIONS:NH2,17.026549105,OH,18.0105647
##BS2G
#crosslinker:SymetricSingleAminoAcidRestrictedCrossLinker:Name:BS2G;MASS:96.02112055;LINKEDAMINOACIDS:K,S,T,Y,nterm;MODIFICATIONS:NH2,17.026549105,OH,18.0105647,LOOP,0
##============================
asymetric cross-linker (currently modifications need to be specified separately)
##SDA
#
crosslinker:AsymetricSingleAminoAcidRestrictedCrossLinker:Name:SDA;MASS:82.0413162600906;FIRSTLINKEDAMINOACIDS:*;SECONDLINKEDAMINOACIDS:K,S,Y,T,nterm

##################
##Modifications
modifications are possible to be defined as three types:
fixed: every aminoacid is modified
variable: peptides containing the aminoacids will be searched with and without modification
known: not automatically searched - but enables to defined modification as part of the FASTA-file as fixed or variable modification (e.g. defined histon modification
only on histones without haveing to search them everywhere).
linear: peptides with that modification will only be searched as linear peptides (not part of an cross-link)
##
Format is:
modification:(fixed|variable|known)::SYMBOL:(how is the modification represented);MODIFIED:[aminoaid];MASS:[mass of the modified amino acid]
Symbol: peptides will be reported with the modification as part of the
sequence the symbol to represent the modified amino acid
Ccm for Carboxyamidomethylation of Cysteine
MODIFIED: the amni-acid to be modified (e.g. C)
MASS: the total mass of the modified amino acid
(This format is also used to define amino-acid substitution)
##
Alternativly modifications that apply to several aminoacids can also be defined as

```
##        modification:variable::SYMBOLEXT:[extension];MODIFIED:[amino-acids];DELTAMASS:[mass-difference]
##  SYMBOLEXT:   What will be appended to the amino-acid to denote this modification (E.g. ox for oxidation)
##  MODIFIED:    A list of aminoa cids that can have this modification
##  DELTAMASS:   the mass diference between the modified and teh undmodified version of the amino-acid.
##
##========================
##--Fixed Modifications
#modification:fixed::SYMBOLEXT:cm;MODIFIED:C;DELTAMASS:57.021464

##========================
##--Variable Modifications
##Mox = 131.040485 + 15.99491
modification:variable::SYMBOLEXT:ox;MODIFIED:M;DELTAMASS:15.99491463
#modification:variable::SYMBOLEXT:bs3oh;MODIFIED:K,S,T,Y;DELTAMASS:156.0786347
#modification:variable::SYMBOLEXT:bs3nh2;MODIFIED:K,S,T,Y;DELTAMASS:155.094619105
# modification:variable::SYMBOLEXT:ox;MODIFIED:M,Q,N;DELTAMASS:15.99491463
# modification:variable::SYMBOL:Mox;MODIFIED:M;MASS:147.035395


##========================
##--linear Modifications
## peptides containing the given amino-acids will be searched with and without
## the modification - BUT the modified version will not be searched as part of a
## cross-link
# modification:linear::SYMBOLEXT:bs3oh;MODIFIED:K,S,T,Y;DELTAMASS:156.0786347
# modification:linear::SYMBOLEXT:bs3nh2;MODIFIED:K,S,T,Y;DELTAMASS:155.094619105


###################
## Digest
##Tryptic digest
#digestion:PostAAConstrainedDigestion:DIGESTED:K,R;ConstrainingAminoAcids:P;NAME=Trypsin
digestion:PostAAConstrainedDigestion:DIGESTED:K,R;ConstrainingAminoAcids:;NAME=Trypsin\P
#digestion:PostAAConstrainedDigestion:DIGESTED:K;ConstrainingAminoAcids:P;NAME=LysC
##No Digestion e.g. for Synthetic Peptide
#digestion:NoDigestion:




###########################################################################################
###########
##Fragment match settings

####################
## Non-Lossy Fragments to consider
fragment:BIon
fragment:YIon
#fragment:CIon
#fragment:ZIon
#fragment:AIon
#fragment:XIon
```

peptide ion should always be enabled, as otherwise no standard cross-linked fragments will be matched - also needed for precoursor-fragment matches
fragment:PeptideIon
enables double fragmentation with in one fragment but also fragmentation events on both peptides
#fragment:BLikeDoubleFragmentation;ID:4

##################
##Losses
Water
loss:AminoAcidRestrictedLoss:NAME:H20;aminoacids:S,T,D,E;MASS:18.01056027;cterm
Amonia
loss:AminoAcidRestrictedLoss:NAME:NH3;aminoacids:R,K,N,Q;MASS:17.02654493;nterm
CH3SOH from Mox
#loss:AminoAcidRestrictedLoss:NAME:CH3SOH;aminoacids:Mox;MASS:63.99828547
##AIons as loss from BIons
when defiend as loss the matched fragments will have less impact on the score then matching A-Ions
loss:AIonLoss
##crosslinker modified fragment (fragmentation of the cross-linker petide bound)
#loss:CrosslinkerModified

###################
include linear matches
EVALUATELINEARS:true

####################
Generally lossy fragmenst will have a smaller impact on subscores then non-lossy versions of a fragment.
But some subscores (anything called conservative) considere a fragment observed even if n neutral losses for that fragment where observed but not the fragment itself
this defines how many loses are needed to make a fragment count as observed
ConservativeLosses:3

###################
if this is set to true also fragment matches are reported that are of by 1 dalton
MATCH_MISSING_MONOISOTOPIC:true

###################
how many peaks to consider for alpha-peptide-search
mgcpeaks:10

```
###################
### Candidate selection
## Scoreing happens in three stages
## alpha candidates are selected and scored
## top n aplpha candidates are taken and all matching beta-candidates will be selected and prescored
## the top X of these are then fully matched and scored
## how many "alpha" peptide candidiates will be considered for finding beta candidates
topmgchits:150
## how many combinations of alpha and beta peptides will be considered for final scoring
topmgxhits:10

##################
## how many misscleavages are considered
missedcleavages:3

####################
## define a minimum peptide length (default 2)
MINIMUM_PEPTIDE_LENGTH:6


#####################
## IO-settings - for improving the parallel processing it's better to do some buffering
## this reduces the time threads potentially have to wait for spectra to be searched (BufferInput)
## or to be written out (BufferOutput).
BufferInput:100
BufferOutput:100






#####################
## Only write out the top match per spectrum
TOPMATCHESONLY:true

#####################
## maximum mass of a peptide to be considered for fragmentation
#MAXPEPTIDEMASS:8000

#####################
## some limits for generating modified peptides
MAX_MODIFICATION_PER_PEPTIDE:5
MAX_MODIFIED_PEPTIDES_PER_PEPTIDE:20

####################
##If the top-match for a spectra has a score lower than this, the spectra and all of its matches are not reported
#MINIMUM_TOP_SCORE:0
```

```
##########################################
## what fragment tree to use
## default: the default tree
## FU: uses a fastutil based implementation of the fragmenttree and conservea lot of memory doing so.
## searching a few hunderd proteins is then possible with just 8GB
FRAGMENTTREE:FU


##########################################
## we need the run name and scan number for a spectrum
## but mgf-files have that info (if at all) in the TITLE line
## and it is not exactly defined how that is stored
## some mgf-files that we have encountered are already regognised for others
## the following to regular expressions can be defined to read out scan number and run
## if both are supplied these will be first tried before the internal automatic will be used
## the scan number and the raw file need to be in the first capturing group
## Example:
## the mgf contains headers like:
## TITLE= Elution from: -1.0 to -1.0 period:  experiment:  cycles:  precIntensity: -1.0 RawFile: myrawfile
FinneganScanNumber: 14846
## then the regular expressions should be defined as
## SCAN_RE: .*FinneganScanNumber:\s*([0-9]*)\s*
## RUN_RE: .*RawFile:\s*(.*)FinneganScanNumber:
##
## XiSearch comes with a range of know regulare mgf-title formats but there are a lot
## more formats out there. So if you encounter an error that the file is not known try this.
##
#SCAN_RE:
#RUN_RE:



##########################################
## for a fragment up to how many neutral losses for that fragment are considered
#MAXTOTALLOSSES:

##########################################
## for each type of loss up to how often is that considered for a single fragment
#MAXLOSSES:

##########################################
## if a spectrum comes with a list of predefined masses for the peptides
## take these as the exclusively accepted masses or just give them priorety?
#XL_Peptide_Mass_Candidates_Exclusive:false


#########################################
## the normalized score by default ignores subscores that are not defined
## with this setting the miossing score can be replaced by a different score
## this replacement would be replacement for the normalized score and the most
```

```
## sensible replacement I coudl see would be "1".
##
#normalizerml_defaultsubscorevalue:1

#########################################
##InputFIlter that modify the spectra before search
##
##DENOISE
## very beta - don't use
## denoise the spectra prior search (default top 20 peaks per 100 Da are kept
#FILTER:denoise:peaks:15;window:100

#########################################
## consider also matches to a precursor mass that
## are up to n Da (actually n*1.00335Da) lighter
## this would account for missing isotope peaks in the MS1
missing_isotope_peaks:2
```

MaxQuant

Parameter	Value
Version	2.0.3.0
User name	aghippler
Machine name	IBBPHI41
Date of writing	03/12/2024 21:40:24
Include contaminants	True
PSM FDR	0.01
PSM FDR Crosslink	0.01
Protein FDR	0.01
Site FDR	0.01
Use Normalized Ratios For Occupancy	True
Min. peptide Length	6
Min. score for unmodified peptides	0
Min. score for modified peptides	40
Min. delta score for unmodified peptides	0
Min. delta score for modified peptides	6
Min. unique peptides	0
Min. razor peptides	1
Min. peptides	1
Use only unmodified peptides and	True
Modifications included in protein quantification	Oxidation (M);Acetyl (Protein N-term)
Peptides used for protein quantification	Razor
Discard unmodified counterpart peptides	True
Label min. ratio count	2
Use delta score	False
iBAQ	False
iBAQ log fit	False
Match between runs	False
Find dependent peptides	False
Fasta file	D:\Crosslink-Paper\Uniprot_Chlamy_for_Cross-Linking_2023.fasta
Decoy mode	revert
Include contaminants	True
Advanced ratios	True
Fixed andromeda index folder
Combined folder location
Second peptides	True
Stabilize large LFQ ratios	True
Separate LFQ in parameter groups	False
Require MS/MS for LFQ comparisons	True
Calculate peak properties	False
Main search max. combinations	200
Advanced site intensities	True
Write msScans table	False
Write msmsScans table	True
Write ms3Scans table	True
Write allPeptides table	True
Write mzRange table	True
Write DIA fragments table	False
Write DIA fragments quant table	False
Write pasefMsmsScans table	True
Write accumulatedMsmsScans table	True

Max. peptide mass [Da] 4600
Min. peptide length for unspecific search 8
Max. peptide length for unspecific search 25
Razor protein FDR True
Disable MD5 False
Max mods in site table 3
Match unidentified features False
Epsilon score for mutations
Evaluate variant peptides separately True
Variation mode None
MS/MS tol. (FTMS) 20 ppm
Top MS/MS peaks per Da interval. (FTMS) 12
Da interval. (FTMS) 100
MS/MS deisotoping (FTMS) True
MS/MS deisotoping tolerance (FTMS) 7
MS/MS deisotoping tolerance unit (FTMS) ppm
MS/MS higher charges (FTMS) True
MS/MS water loss (FTMS) True
MS/MS ammonia loss (FTMS) True
MS/MS dependent losses (FTMS) True
MS/MS recalibration (FTMS) False
MS/MS tol. (ITMS) 0.5 Da
Top MS/MS peaks per Da interval. (ITMS) 8
Da interval. (ITMS) 100
MS/MS deisotoping (ITMS) False
MS/MS deisotoping tolerance (ITMS) 0.15
MS/MS deisotoping tolerance unit (ITMS) Da
MS/MS higher charges (ITMS) True
MS/MS water loss (ITMS) True
MS/MS ammonia loss (ITMS) True
MS/MS dependent losses (ITMS) True
MS/MS recalibration (ITMS) False
MS/MS tol. (TOF) 40 ppm
Top MS/MS peaks per Da interval. (TOF) 10
Da interval. (TOF) 100
MS/MS deisotoping (TOF) True
MS/MS deisotoping tolerance (TOF) 0.01
MS/MS deisotoping tolerance unit (TOF) Da
MS/MS higher charges (TOF) True
MS/MS water loss (TOF) True
MS/MS ammonia loss (TOF) True
MS/MS dependent losses (TOF) True
MS/MS recalibration (TOF) False
MS/MS tol. (Unknown) 20 ppm
Top MS/MS peaks per Da interval. (Unknown) 12
Da interval. (Unknown) 100
MS/MS deisotoping (Unknown) True
MS/MS deisotoping tolerance (Unknown) 7
MS/MS deisotoping tolerance unit (Unknown) ppm
MS/MS higher charges (Unknown) True
MS/MS water loss (Unknown) True
MS/MS ammonia loss (Unknown) True
MS/MS dependent losses (Unknown) True

MS/MS recalibration (Unknown)	False
Site tables	Oxidation (M)Sites.txt

```
<PersistedProcessingRoutine
xmlns="http://schemas.datacontract.org/2004/07/MassSpecStudio.Core.Processing"
xmlns:i="http://www.w3.org/2001/XMLSchema-instance"><Name>Crosslinked Peptide
Search</Name><TypeId>crimp2-search-routine-04df2620-5285-482d-825a-
71bde7be45a8</TypeId><Date>2024-04-16T12:23:22.298534+02:00</Date><ProcessingSteps
xmlns:a="http://schemas.microsoft.com/2003/10/Serialization/Arrays"><a:KeyValueOfstringPersist
edProcessingStepHaK2COcM><a:Key>Cross-Link Search Parameters</a:Key><a:Value><Name>Cross-Link
Peptide Search
Settings</Name><Type>CrossLink.Processing.Steps.CrossLinkRoutineParameters</Type><Parameters><
PersistedProcessingParameter><Key>AggregationTarget</Key><Value>Dataset</Value></PersistedProc
essingParameter><PersistedProcessingParameter><Key>NormalizeFinalRTs</Key><Value>True</Value><
/PersistedProcessingParameter><PersistedProcessingParameter><Key>ConflictResolution</Key><Valu
e>True</Value></PersistedProcessingParameter><PersistedProcessingParameter><Key>PropagateDB</K
ey><Value>State
Group</Value></PersistedProcessingParameter><PersistedProcessingParameter><Key>TimeSplits</Key
><Value>1</Value></PersistedProcessingParameter><PersistedProcessingParameter><Key>ExtractXICs
</Key><Value>False</Value></PersistedProcessingParameter></Parameters></a:Value></a:KeyValueOf
stringPersistedProcessingStepHaK2COcM><a:KeyValueOfstringPersistedProcessingStepHaK2COcM><a:Ke
y>Fast-Tracked Export</a:Key><a:Value><Name>Fast-Track
Exporter</Name><Type>CrossLink.Processing.Steps.OptionalExportStep</Type><Parameters><Persiste
dProcessingParameter><Key>CSMExportPath</Key><Value/></PersistedProcessingParameter><Persisted
ProcessingParameter><Key>RemoveCSMDecoys</Key><Value>True</Value></PersistedProcessingParamete
r><PersistedProcessingParameter><Key>CSMQThreshold</Key><Value>0.05</Value></PersistedProcessi
ngParameter><PersistedProcessingParameter><Key>UPPExportPath</Key><Value/></PersistedProcessin
gParameter><PersistedProcessingParameter><Key>RemoveUPPDecoys</Key><Value>False</Value></Persi
stedProcessingParameter><PersistedProcessingParameter><Key>UPPQThreshold</Key><Value>0.05</Val
ue></PersistedProcessingParameter><PersistedProcessingParameter><Key>URPExportPath</Key><Value
/></PersistedProcessingParameter><PersistedProcessingParameter><Key>RemoveURPDecoys</Key><Valu
e>False</Value></PersistedProcessingParameter><PersistedProcessingParameter><Key>URPQThreshold
</Key><Value>0.05</Value></PersistedProcessingParameter><PersistedProcessingParameter><Key>Pro
teinsExportPath</Key><Value/></PersistedProcessingParameter><PersistedProcessingParameter><Key
>RemoveProteinDecoys</Key><Value>False</Value></PersistedProcessingParameter><PersistedProcess
ingParameter><Key>ProteinQThreshold</Key><Value>0.05</Value></PersistedProcessingParameter><Pe
rsistedProcessingParameter><Key>PPIExportPath</Key><Value/></PersistedProcessingParameter><Per
sistedProcessingParameter><Key>RemovePPIDecoys</Key><Value>True</Value></PersistedProcessingPa
rameter><PersistedProcessingParameter><Key>PPIQThreshold</Key><Value>0.05</Value></PersistedPr
ocessingParameter></Parameters></a:Value></a:KeyValueOfstringPersistedProcessingStepHaK2COcM><
a:KeyValueOfstringPersistedProcessingStepHaK2COcM><a:Key>Database
Reducer</a:Key><a:Value><Name>Linear Peptide
Search</Name><Type>CrossLink.Processing.Steps.PeptideDatabaseCreators.DirectProteinFragmentSea
rch</Type><Parameters><PersistedProcessingParameter><Key>ProteinChunkSize</Key><Value>50</Valu
e></PersistedProcessingParameter><PersistedProcessingParameter><Key>SkipY1Fragments</Key><Valu
e>True</Value></PersistedProcessingParameter><PersistedProcessingParameter><Key>IncludeComplem
entFragments</Key><Value>True</Value></PersistedProcessingParameter><PersistedProcessingParame
ter><Key>IncludeXLSpecificProductIons</Key><Value>False</Value></PersistedProcessingParameter>
<PersistedProcessingParameter><Key>IncludeNeutralLossFragments</Key><Value>False</Value></Pers
istedProcessingParameter><PersistedProcessingParameter><Key>DenoiseSpectra</Key><Value>False</
Value></PersistedProcessingParameter><PersistedProcessingParameter><Key>ValidateFreeFragments<
/Key><Value>True</Value></PersistedProcessingParameter><PersistedProcessingParameter><Key>Frag
mentCountThreshold</Key><Value>2</Value></PersistedProcessingParameter><PersistedProcessingPar
ameter><Key>MIPOnly</Key><Value>True</Value></PersistedProcessingParameter><PersistedProcessin
gParameter><Key>DeIsotopedData</Key><Value>True</Value></PersistedProcessingParameter><Persist
edProcessingParameter><Key>DigestionName</Key><Value>Trypsin (K/R
only)</Value></PersistedProcessingParameter><PersistedProcessingParameter><Key>CreateDecoys</K
ey><Value>True</Value></PersistedProcessingParameter><PersistedProcessingParameter><Key>MinSiz
e</Key><Value>6</Value></PersistedProcessingParameter><PersistedProcessingParameter><Key>MaxSi
ze</Key><Value>60</Value></PersistedProcessingParameter><PersistedProcessingParameter><Key>Squ
ashIsoformPeptides</Key><Value>True</Value></PersistedProcessingParameter><PersistedProcessing
Parameter><Key>ChargeStatesString</Key><Value>1,2</Value></PersistedProcessingParameter><Persi
stedProcessingParameter><Key>FragmentCountThreshold</Key><Value>2</Value></PersistedProcessing
Parameter><PersistedProcessingParameter><Key>PercentEThreshold</Key><Value>99</Value></Persist
edProcessingParameter><PersistedProcessingParameter><Key>StorageFactoryType</Key><Value>Defaul
t</Value></PersistedProcessingParameter></Parameters></a:Value></a:KeyValueOfstringPersistedPr
ocessingStepHaK2COcM><a:KeyValueOfstringPersistedProcessingStepHaK2COcM><a:Key>Cross-Linked
Peptide Generator</a:Key><a:Value><Name>Cross-linked Peptide DB
Creation</Name><Type>CrossLink.Processing.Steps.CrossLinkedPeptideGenerators.RTIterativeMassGr
oupedPairing</Type><Parameters><PersistedProcessingParameter><Key>IncludeNH3LossPrecursors</Ke
y><Value>False</Value></PersistedProcessingParameter><PersistedProcessingParameter><Key>Includ
```

```
eH2OLossPrecursors</Key><Value>False</Value></PersistedProcessingParameter><PersistedProcessin
gParameter><Key>SearchXLDE</Key><Value>False</Value></PersistedProcessingParameter><PersistedP
rocessingParameter><Key>AlphaPercentEThreshold</Key><Value>25</Value></PersistedProcessingPara
meter><PersistedProcessingParameter><Key>TopN</Key><Value>10</Value></PersistedProcessingParam
eter><PersistedProcessingParameter><Key>UseReportedCharge</Key><Value>True</Value></PersistedP
rocessingParameter><PersistedProcessingParameter><Key>MinMassRange</Key><Value>400</Value></Pe
rsistedProcessingParameter><PersistedProcessingParameter><Key>MaxMassRange</Key><Value>1600</V
alue></PersistedProcessingParameter><PersistedProcessingParameter><Key>MinCharge</Key><Value>3
</Value></PersistedProcessingParameter><PersistedProcessingParameter><Key>MaxCharge</Key><Valu
e>8</Value></PersistedProcessingParameter><PersistedProcessingParameter><Key>InSourceFragmenta
tion</Key><Value>False</Value></PersistedProcessingParameter><PersistedProcessingParameter><Ke
y>MaxRealIsotopes</Key><Value>4</Value></PersistedProcessingParameter><PersistedProcessingPara
meter><Key>NegativeIsotopesToCheck</Key><Value>1</Value></PersistedProcessingParameter></Param
eters></a:Value></a:KeyValueOfstringPersistedProcessingStepHaK2COcM><a:KeyValueOfstringPersist
edProcessingStepHaK2COcM><a:Key>Crosslink Fragment Search</a:Key><a:Value><Name>Standard XLink
Processing</Name><Type>CrossLink.Processing.Steps.CrossLinkPeptideSearch.DefaultProcessor</Typ
e><Parameters><PersistedProcessingParameter><Key>NumFragEventsAllowed</Key><Value>2</Value></P
ersistedProcessingParameter><PersistedProcessingParameter><Key>SearchNeutralLossFragments</Key
><Value>False</Value></PersistedProcessingParameter><PersistedProcessingParameter><Key>Denoise
Spectra</Key><Value>False</Value></PersistedProcessingParameter><PersistedProcessingParameter>
<Key>ExcludeY1s</Key><Value>False</Value></PersistedProcessingParameter><PersistedProcessingPa
rameter><Key>FragmentIntensityCutoff</Key><Value>25</Value></PersistedProcessingParameter><Per
sistedProcessingParameter><Key>FragmentCountThreshold</Key><Value>2</Value></PersistedProcessi
ngParameter></Parameters></a:Value></a:KeyValueOfstringPersistedProcessingStepHaK2COcM><a:KeyV
alueOfstringPersistedProcessingStepHaK2COcM><a:Key>FDR
Reduction</a:Key><a:Value><Name>Spectrum Match FDR
Estimator</Name><Type>CrossLink.Processing.Steps.CrimpFDR</Type><Parameters><PersistedProcessi
ngParameter><Key>FalseDiscoveryRate</Key><Value>0.05</Value></PersistedProcessingParameter></P
arameters></a:Value></a:KeyValueOfstringPersistedProcessingStepHaK2COcM><a:KeyValueOfstringPer
sistedProcessingStepHaK2COcM><a:Key>CSM Flagging Controller</a:Key><a:Value><Name>Flagging
Control</Name><Type>CrossLink.Processing.Steps.RunResultInspector</Type><Parameters><Persisted
ProcessingParameter><Key>MinContinuousFragments</Key><Value>3</Value></PersistedProcessingPara
meter></Parameters></a:Value></a:KeyValueOfstringPersistedProcessingStepHaK2COcM><a:KeyValueOf
stringPersistedProcessingStepHaK2COcM><a:Key>MS1 Mass Accuracy</a:Key><a:Value><Name>MS1
Accurate Mass
Selector</Name><Type>MassSpecStudio.Processing.Steps.AccurateMassSelector</Type><Parameters><P
ersistedProcessingParameter><Key>MassToleranceOption</Key><Value>PPM</Value></PersistedProcess
ingParameter><PersistedProcessingParameter><Key>MassTolerance</Key><Value>10</Value></Persiste
dProcessingParameter></Parameters></a:Value></a:KeyValueOfstringPersistedProcessingStepHaK2COc
M><a:KeyValueOfstringPersistedProcessingStepHaK2COcM><a:Key>MS2 Mass
Accuracy</a:Key><a:Value><Name>MS2 Accurate Mass
Selector</Name><Type>MassSpecStudio.Processing.Steps.AccurateMassSelector</Type><Parameters><P
ersistedProcessingParameter><Key>MassToleranceOption</Key><Value>PPM</Value></PersistedProcess
ingParameter><PersistedProcessingParameter><Key>MassTolerance</Key><Value>20</Value></Persiste
dProcessingParameter></Parameters></a:Value></a:KeyValueOfstringPersistedProcessingStepHaK2COc
M><a:KeyValueOfstringPersistedProcessingStepHaK2COcM><a:Key>Peptide Elution
Time</a:Key><a:Value><Name>Elution Time
Selector</Name><Type>MassSpecStudio.Processing.Steps.ElutionTimeSelector</Type><Parameters><Pe
rsistedProcessingParameter><Key>ElutionWidth</Key><Value>0.5</Value></PersistedProcessingParam
eter></Parameters></a:Value></a:KeyValueOfstringPersistedProcessingStepHaK2COcM><a:KeyValueOfs
tringPersistedProcessingStepHaK2COcM><a:Key>XIC Extraction</a:Key><a:Value><Name>Basic XIC
Generator</Name><Type>MassSpecStudio.Processing.Steps.BasicXicExtractor</Type><Parameters><Per
sistedProcessingParameter><Key>MassToleranceOption</Key><Value>PPM</Value></PersistedProcessin
gParameter><PersistedProcessingParameter><Key>MassTolerance</Key><Value>10</Value></PersistedP
rocessingParameter></Parameters></a:Value></a:KeyValueOfstringPersistedProcessingStepHaK2COcM>
<a:KeyValueOfstringPersistedProcessingStepHaK2COcM><a:Key>MS1
Extraction</a:Key><a:Value><Name>Basic Spectrum
Generator</Name><Type>MassSpecStudio.Processing.Steps.BasicSpectrumSelection</Type><Parameters
><PersistedProcessingParameter><Key>MZLowerOffset</Key><Value>-
2</Value></PersistedProcessingParameter><PersistedProcessingParameter><Key>MZUpperOffset</Key>
<Value>5</Value></PersistedProcessingParameter><PersistedProcessingParameter><Key>QueryUsingMo
noIsotopic</Key><Value>True</Value></PersistedProcessingParameter><PersistedProcessingParamete
r><Key>ExtractionOption</Key><Value>Summed</Value></PersistedProcessingParameter></Parameters>
</a:Value></a:KeyValueOfstringPersistedProcessingStepHaK2COcM><a:KeyValueOfstringPersistedProc
essingStepHaK2COcM><a:Key>XIC Peak Picking</a:Key><a:Value><Name>XIC Peak
Detector</Name><Type>MassSpecStudio.Processing.Steps.ChromatographicPeakDetection</Type><Param
eters><PersistedProcessingParameter><Key>PeakToBackgroundRatio</Key><Value>2</Value></Persiste
dProcessingParameter></Parameters></a:Value></a:KeyValueOfstringPersistedProcessingStepHaK2COc
M><a:KeyValueOfstringPersistedProcessingStepHaK2COcM><a:Key>MS1 Peak
Picking</a:Key><a:Value><Name>Basic m/z Peak
Detector</Name><Type>MassSpecStudio.Processing.Steps.BasicSpectralPeakDetection</Type><Paramet
```

ers><PersistedProcessingParameter><Key>PeakToBackgroundRatio</Key><Value>0.1</Value></Persiste
dProcessingParameter></Parameters></a:Value></a:KeyValueOfstringPersistedProcessingStepHaK2COc
M><a:KeyValueOfstringPersistedProcessingStepHaK2COcM><a:Key>MS2 Peak
Picking</a:Key><a:Value><Name>Basic m/z Peak
Detector</Name><Type>MassSpecStudio.Processing.Steps.BasicSpectralPeakDetection</Type><Paramet
ers><PersistedProcessingParameter><Key>PeakToBackgroundRatio</Key><Value>0.05</Value></Persist
edProcessingParameter></Parameters></a:Value></a:KeyValueOfstringPersistedProcessingStepHaK2CO
cM><a:KeyValueOfstringPersistedProcessingStepHaK2COcM><a:Key>XIC Smoothing</a:Key><a:Value
i:nil="true"/></a:KeyValueOfstringPersistedProcessingStepHaK2COcM><a:KeyValueOfstringPersisted
ProcessingStepHaK2COcM><a:Key>MS1 Smoothing</a:Key><a:Value
i:nil="true"/></a:KeyValueOfstringPersistedProcessingStepHaK2COcM><a:KeyValueOfstringPersisted
ProcessingStepHaK2COcM><a:Key>MS2 Smoothing</a:Key><a:Value
i:nil="true"/></a:KeyValueOfstringPersistedProcessingStepHaK2COcM></ProcessingSteps><IsPermane
nt>false</IsPermanent></PersistedProcessingRoutine>
